# Pitfalls in the diagnosis of Gaucher disease in Iraq: A diagnostic experience from a developing country

**DOI:** 10.12669/pjms.37.3.2930

**Published:** 2021

**Authors:** Rabab Farhan Thejeal, Saja Baheer Abdul wahhab, Nebal Waill Saadi

**Affiliations:** 1 Rabab Farhan Thejeal Assistant Professor, College of Medicine-University of Baghdad, Pediatric Department, Pediatric Gastroenterology, Children Welfare Teaching Hospital, Medical City Complex, Baghdad, Iraq; 2 Saja Baheer Abdul Wahhab Pediatrician-C.A.B.P., Metabolic Clinic, Children Welfare Teaching Hospital, Baghdad, Iraq; 3 Nebal Waill Saadi Assistant Professor, College of Medicine, University of Baghdad, Pediatric Neurology, Children Welfare Teaching Hospital, Baghdad, Iraq.

**Keywords:** Gaucher disease, Diagnostic errors, Developing country

## Abstract

**Background and Objectives:**

Gaucher disease (GD) is a rare hereditary disorder caused by deficiency of the lysosomal enzyme β-glucocerebrosidase. An early and definitive diagnosis minimizes the sequelae of misdiagnoses, and unnecessary and invasive diagnostic procedures.

**Methods:**

A cross-sectional descriptive study was conducted in the period from June to August, 2018, to analysing data of thirteen patients, retrospectively. They presented to the gastrointestinal and metabolic clinics in Children Welfare Teaching Hospital in Iraq, and had wrong and delayed diagnosis of GD.

**Results:**

Two groups of patients were identified, based on diagnosis by enzymatic assay (considering the test positive when the enzyme level is below the normal value); those who had false positive (low level of the enzyme) result and received enzyme replacement therapy for long time, and those who had false negative (normal level of the enzyme) results which caused delay in their diagnosis and treatment. Two main factors that misled the diagnosis were identified.

**Conclusion:**

Each patient with Gaucher disease need to be approached by taking a thorough history, a proper clinical examination, and then by being investigated, accordingly. Biomarkers and molecular genetic studies are more accurate and solid additional tools, to the enzymatic assays on dried blood sample (DBS).

## INTRODUCTION

Gaucher disease (GD) is a rare, autosomal recessive genetic disease caused by mutations in the glucocerebrosidase (GBA1) gene, on chromosome 1 (1q21). This leads to a markedly decreased activity of the lysosomal enzyme glucocerebrosidase.^1^ The most common presenting features are anemia, splenomegaly, thrombocytopenia and bone pain. Diagnosis is made by clinical history, physical examination and laboratory tests, and is confirmed by a specific blood test showing deficiency of the enzyme activity. Additionally, the genetic study confirm the genetic mutation in GBA1 gene.^2^ Erroneous diagnostic decisions can lead to additional multiple hospital visits or stay, clinical deterioration, inappropriate therapy and unnecessary procedures’ complications, delayed diagnosis of the original disease and psychological trauma by the false beliefs of having the disease. The aim of this study was to evaluate the possible pitfalls in diagnosing GD in Iraqi patients, while correlating the clinical and laboratory results.

## METHODS

A descriptive study was conducted in Children Welfare Teaching Hospital, Baghdad, which is one of the biggest tertiary children hospitals in Iraq, in the period from June to August, 2018. Once the confirmative biomarker and genetic tests became accessible in laboratories abroad, GD children were retested. Out of 66 patients, who were initially diagnosed with GD according to enzymatic level evaluation, 11 showed normal genetic and biochemical markers. Those were finally exempted from the total number of GD patients and from the management program. Additional two patients who had quite suggestive picture of GD clinically and positive family history (first degree relatives) of GD, and showed normal enzymatic analysis, were re-evaluated by molecular and biomarker tests, both of which confirmed the diagnosis of GD. So, only 57 patients with confirmed GD diagnosis remained. The medical records of the 13 patients were reviewed retrospectively. The concept of laboratory and clinical false positive and false negative cases of GD was suggested. Demographics, clinical and laboratory data were extracted, including radiological assessment by skeletal survey. All the results were described and the diagnostic pitfalls were discussed. This study was approved by the Local Ethical Committee in Children Welfare Teaching Hospital (IRB: 31, Dated: 1^st^ of May, 2018).

For all thirteen patients, the usual practice was to use blood samples for Lysosomal enzyme assays via filter cards of dried blood spot (DBS) (Whatman 903 cards), which were provided by Genzyme / Sanofi Company (Iraq). Two millilitres of blood were collected in EDTA tube and dropped into the cards, that were delivered through DHL to ICLD Hamburge (Germany), Princess Haya Lab Biotechnology Centers (Jordan), and Centogen “The Rare Disease Company” (Germany). They were all analysed by tandem mass spectrometry method, which is considered as the most accurate diagnostıc procedure on DBS samples.^3^,^4^ Moreover, specific filter cards were offered by Genzyme/ Sanofi company to send blood samples that were tested for the molecular profile of GBA gene. These were performed in Centogen “The Rare Disease Company” (Germany) and Archimed Life Science GmbH (Austria), by amplicon - based next generation sequencing approach covering the entire coding region of the GBA gene. Additional one patient had genetic testing performed in Iraq, using PCR - based technique to test for the eight common mutations of the GBA gene.

## RESULTS

The studied patients were thirteen. They were divided into two groups. Table-I, II & III showed the clinical manifestations, laboratory results, treatment and follow up, respectively, for each patient in both groups. The first group is composed of eleven patients (1-11) who had false positive enzymatic testing. They have been treated with enzyme replacement therapy for long period of time. While the second group is composed of two patients (12 & 13) who had strong clinical evidence of GD, and whose first degree relatives have been diagnosed with GD and were included in the cohort that has been managed in Children Welfare Teaching Hospital. Their enzyme activity showed normal results on DBS. Re-analysis using Lyso GB1 and molecular tests, were necessitated and both confirmed the diagnosis.

**Table-I:** Clinical characteristics of the studied patients.

Patients	Gender	Age of onset (months)	Age of diagnosis (months)	Clinical presentation
1	Male	138	146	Fever, Hepatosplenomegaly
2	Male	32	34	Hepatosplenomegaly
3	Male	9	36	Growth Retardation, hepatomegaly
4	Male	12	36	Psychomotor and speech delay, convulsions, short stature, Hepatosplenomegaly
5	Male	36	60	Bleeding tendency, pallor, splenomegaly
6	Female	21	57	Pallor, upper GI bleeding, Hepatosplenomegaly
7	Female	1	5	Psychomotor delay, pallor, visual and speech problem, microcephaly and hypotonia
8	Male	66	80	Pallor, Hepatosplenomegaly, leg edema, ascites
9	Female	108	127	Pallor, Hepatosplenomegaly
10	Female	96	108	Pallor, Hepatosplenomegaly
11	Female	1	14	Psychomotor delay, strabismus, hypotonia, Hepatosplenomegaly
12	Female	36	45	Pallor, splenomegaly
13	Male	27	48	Pallor, splenomegaly

**Table-II:** Laboratory characteristics of the studied patients.

Patients	Haemoglobin (g/dl)*	Platelets (x103/ul)**	Liver Function Tests***	Skeletal Survey†	Enzyme assay	Molecular study
1	10.4	345	Abnormal	Normal	135.9‡	Negative◊
2	11.5	120	Normal	Normal	183‡	Negative◊
3	10.2	627	Abnormal	Normal	185.4‡	Negative∞
4	11.2	221	Normal	Normal	64.7‡	Negative◊
5	9.6	70	Normal	Normal	197.2‡	Negative◊
6	6.6	52	Normal	Normal	192.4‡	Negative◊
7	11.1	183	Normal	Normal	152.7‡	Negative€
8	9.6	75	Abnormal	Normal	3.5¥	Negative◊
9	5.8	30	Normal	Normal	18.37‡	Negative◊
10	9.9	97	Normal	Normal	18.98‡	Negative◊
11	7.5	70	Abnormal	Normal	104.36‡	Negative∞
12	8.8	130	Normal	Normal	2.5©	Positive∞
13	11.1	174	Normal	Normal	4.8©	Positive∞

* Hb normal values (12-18) g/dl,

** Platelets normal values (145-450 x10^3^/ul),

*** Liver Function Tests include total serum bilirubin, liver enzymes, coagulation profile and total serum protein.

† Skeletal survey regarded as normal due to the absence of signs of Gaucher disease like growth retardation in children, osteopenia, lytic lesions, pathologic fractures, bone pain, osteonecrosis, cortical and medullary infarcts and evidence of bone crises.

‡ Normal values 200 – 2000 Pmol/spot*20hour

¥ Normal Values 5 – 47nmol/ml/hour

∞ PCR amplification and sequencing of all coding exons and flanking and intronic regions in *GBA* gene.

€ PCR and reverse hybridization of 8 common mutations of *GBA* gene.

© Normal cut off values > 1.5 umol/L/hour

◊ NGS-Illumina for all GBA gene mutations.

**Table-III:** Treatment and follow up of the studied patients.

Patients	Treatment duration (months) ^¤^	Follow up
1	28	Early complete response, absent relapse during treatment unavailability
2	43	Early complete response, absent relapse during treatment unavailability
3	44	Recurrent hypoglycemia, elevated triglycerides and uric acid
4	60	Recurrent hypoglycemia, no response to treatment (organomegaly)
5	21	No response to treatment (organomegaly) and low Platelets count
6	24	No response to treatment (organomegaly)
7	24	Signs not related to Gaucher disease
8	37	Symptoms of chronic liver disease, No response to treatment (organomegaly)
9	26	No response to treatment (organomegaly)
10	24	No response to treatment (organomegaly)
11	2	No response to treatment (organomegaly)
12	-	Elevated Lyso GL1 and positive family history of Gaucher disease
13	-	Elevated Lyso GL1 and positive family history of Gaucher disease

¤ Treatment was ERT (Imiglucerase) 60 IU/kg every 2 weeks

The frequency of distribution of variable physical signs among the studied group is shown in Table-IV. Organomegaly and pallor were the most common clinical signs detected, to be followed by neurological manifestations.

**Table-IV:** Frequency of the clinical features of the studied patients.

Clinical Feature	No. (n= 13)	Percentage (%)
Pallor	8	61.5
Fever	1	7.7
Organomegaly	12	92.3
Growth Retardation	1	7.7
Bleeding Tendency	2	15.4
Neurological Manifestations	3	23.1
Skeletal Manifestation	0	0

The seasonal distribution of the studied patients based on the season when the DBS of each patient was sent abroad for enzymatic assay is shown in Table-V.

**Table-V:** Seasonal distribution of the studied patients.

Season	No. (n= 13)	Percentage (%)
Summer	6	46.2
Spring	4	30.8
Winter	2	15.4
Autumn	1	7.7

## DISCUSSION

Iraq is a country with high consanguinity and intertribal marriages’ rate reaching up to 48%,^5^ a fact that can lead to a higher percentages of inherited disorders, particularly autosomal recessive disorders as GD.^6^ In 2009, a project of testing for lysosomal storage disorders by sending dried blood samples (DBS) to laboratories in Germany / Jordan for enzymes assay, was launched. Since that time, and until writing this report, 95 cases of Gaucher disease were diagnosed and managed in Iraq, of whom 66 were in Children Welfare Teaching Hospital - Baghdad. Assessment of Lyso GB1 and molecular analysis of GBA mutations for Gaucher patients were luckily and smoothly offered on 2017. At that time, re-testing the already diagnosed and treated patients and two additional and suspicious cases (patient 12 & 13), revealed contradicting results.

The patients showed features (Table-I, II, III and IV) that variably matched the clinical and laboratory profile of GD as the following: pallor (patient 5,6,7,8,9,10,12,13), enlarged spleen and/or liver (patient 1,2,3,4,5,6,8,9,10,11,12), bleeding tendency (patient 5, 6), anemia (all patients) and thrombocytopenia (patient 2,5,6,8,9,10,11,12). None of them had any skeletal manifestations. In a list of differential diagnoses set for each case scenario, GD was considered and finally, diagnosis and treatment was governed by the results of enzymatic assay.

Reviewing both groups explored manifestations that cast doubts about GD diagnosis, despite the enzymatic levels seen in DBS tests. Those were: 1) Episodes of hypoglycemia (patient 3 & 4). GD does not cause that serious liver impairment, that result in a state of hypoglycemia in starvation conditions. Nothing was reported in the literature about hypoglycemia as part of the clinical features in GD, unless comorbid with other endocrine or hepatic disorders; 2) Advanced liver disease leading to portal hypertension (patient 8). Liver involvement has been discussed with controversy in the literature, which explored wide spectrum of involvement ranging from sub-clinical involvement to liver cirrhosis, yet, not as prominent presenting features; 3) No satisfied response (as that reported in the literature)^7^ was noted to treatment by enzyme replacement therapy in some patients (patient 5, 6, 9, 10, 11); 4) Unusual neurological features of GD (patient 7). Central nervous system can be affected in some forms of GD. The most common manifestation is impaired saccadic eye movement. Other neurological features have been evidenced, but little is mentioned about psychomotor delay and visual impairment;^8^,^9^ and 5) Family history of sibs with confirmed GD diagnosis in two enzymatically normal patients (patient 12 & 13).

From the above information, two factors that misled the physicians, were surfaced; 1) lack of awareness and proper knowledge, at that time, among non-GD specialists for that rather rare disease, and 2) the enzyme assay test itself can be subjected to false positive and false negative errors.

It is mandatory to disseminate knowledge of GD in the medical community and to encourage the use of diagnostic guidelines, whenever applicable. A number of consensus diagnostic algorithms have been developed to aid non-specialists in the diagnosis of GD, in symptomatic patients.^10^-^12^ These algorithms offer valuable guidance to non-specialists for the differential diagnosis of patients already suspected of having a lysosomal storage disorder.^13^

Despite the fact that presentation with organomegaly, with or without haematological manifestations (which is the manner in all the studied patients except for patient 7), satisfies the diagnostic demands of Gaucher disease and must prompt proceeding with specific investigations, yet, awareness of the typical and classical clinical course of that disease is a prerequisite to avoid diagnostic errors that can result in multiple hospitalizations, deferral of diagnosing the original disease and overloading the patients with unnecessary medications. In the present cohort, positive family history, unusual systematic presentations and metabolic or biochemical disturbances, and unreasonable response to enzyme therapy, should raise suspicions about the diagnosis, without being affected by the abnormal enzymatic analysis and should urge confirmation by additional biomarkers and genetic testing.

Although it is recommended to utilize DBS-based enzyme assay as diagnostic tool for timely identification of patients with lysosomal storage disorders, yet issues related to sample quality might lead to misdiagnosis and / or false positive / negative results. Therefore, it is suggested that coupling investigations, like assessment of biomarkers and molecular genetics, with the enzymatic assay would increase the diagnostic capabilities, especially for suspicious cases.^14^,^15^

Table-V showed that nearly half (46.8%) of the samples were shipped during summer and three fourths during summer and spring. Both seasons are considered, relatively, hot seasons in Iraq.^16^ At the beginning of that diagnostic practice in the country, and with these climatic extremes in temperature and humidity in that hot weather, which might last sometimes up to eight months a year,^16^ mistakes could happen and do happen, like sampling malpractice, while collecting and drying samples, storage and transportation. All these resulted in pre-analytical failure or false low enzyme activity on DBS samples. A fact that was suggested by a previous study, conducted by Stroppianno et al.^17^

Approaching to atypical scenario (as in patient 4 & 7), using a test for a disease with low prevalence, and lacking familiarity with the analytical and clinical accuracy of the test, might all contribute to misdiagnosis.

The outcome of the 13 patients was; patient 1 & 2 had recovered the organomegaly and became asymptomatic. Therefore, infectious illnesses were suspected; Patient three fulfilled the clinical and liver biopsy criteria of glycogen storage disease. Enzyme therapy was discontinued; Patient four outgrew the hypoglycemic attacks by no medication and showed improved developmental profile; Patient five was diagnosed abroad with portal vein thrombosis, operated upon and became well; Contact was lost with patient-6 who had hepatosplenomegaly and esophageal varices; Patient-7 had microcephaly and psychomotor delay, and was advised for high throughput genetic testing, but the family could not afford its cost; Patient 8 & 9 had hepatosplenomegaly, which was later labelled as chronic liver disease. Liver transplantation was planned for one and contact was lost with the other; Patient 10 had splenectomy and diagnosed with sarcoidosis; Patient 11 was thought later to be Niemann Pick Type C; and patient 12 & 13 have been placed on Enzyme replacement therapy and exhibited accepted response (normalization of the hemoglobin and platelet levels and the sizes of liver and spleen).

Despite the presence of local researches that studied the clinical, laboratory and prognostic profile of GD in the Iraqi population, yet, no study has been conducted to address the challenges and opportunities to reduce the diagnostic errors in the local practice.^18^-^24^ This study highlights the misdiagnosis of Gaucher disease in regard of both false positive and false negative tests’ results, and how this causes harm, as the former results in over treatment, delay in the proper diagnosis and the huge impact on the financial aspect of provision of quite costly medicines. While the latter causes delay of effective therapy, increased risk of occurrence of complications and over-investigating the patient with unnecessary tests.

### Limitations of the study:

This study has potential limitations like small sample size and being a single-center based study, which limit the generalizability of the results. Furthermore, it was conducted in a tertiary center, where specific experts are working in that field, therefore the real pitfalls’ frequency cannot be represented, and higher under- or overestimation of Gaucher disease are expected in the practices of physicians of the medical community, who are working in less specialized health-service organizations. Also this study relied on data contained in the medical records and thus, many diagnostic errors were missed like estimation of the costs resulted from this error and lacking the documentation on what the clinician was thinking at the time of diagnosis.

## CONCLUSIONS

Pitfalls in the diagnosis of Gaucher disease do happen. Each patient with Gaucher disease need to be approached by taking a thorough history, a proper clinical examination, and then by being investigated, accordingly. Biomarkers and molecular genetic studies are more accurate and solid additional tools, to the enzymatic assays on dried blood sample (DBS).

### Author’s contribution:

**RFT and SBA:** Design, acquisition of data, analysis, drafting and final approval.

**NWS:** Conception, design, drafting and final approval, accuracy and integrity of the work.
